# Nardilysin: classical functions and new horizons in autoimmunity

**DOI:** 10.3389/fimmu.2026.1858534

**Published:** 2026-08-07

**Authors:** Yashika Choudhary, Ben Ali Moutughimou, Vijay Jagdish Upadhye, Shahnawaz D. Jadeja, Jayvadan Vaishnav

**Affiliations:** 1 Department of Life Sciences, Parul Institute of Applied Sciences, Parul University, Vadodara, Gujarat, India; 2 Department of Microbiology, Parul Institute of Applied Science, Research and Development cell, Parul University, Vadodara, Gujarat, India; 3 Rahodin Limited, Dublin, Ireland

**Keywords:** ADAM17, autoimmunity, ectodomain shedding, Nardilysin, rheumatoid arthritis, T1D, vitiligo, VTCN1

## Abstract

Autoimmune diseases affect 5–10% of the global population, with a prevalence of 13% in women and 7% in men. Despite advances in immunology, the molecular basis of immune dysregulation remains limited. Nardilysin (NRD convertase, NRDc), a zinc-dependent metalloendopeptidase, has been identified as an enzyme involved in this dysregulation. Recent studies have identified NRDc dysregulation as a shared feature across type 1 diabetes (T1D), rheumatoid Arthritis (RA), and vitiligo, though the specific mechanism implicated differs by the disease involved. Reported roles for NRDc include modulating the release of the immune checkpoint protein VTCN1 (B7-H4), modulation of pro-inflammatory cytokines, and activation of ADAM17-mediated release of TNF-α and its receptors. While NRDc is recognized for its role in immune signaling, gene transcription, cell differentiation, proliferation, homeostasis, thermogenesis, and metabolism, a unified framework connecting NRDc’s multifaceted enzymatic activity to autoimmunity is currently lacking. We review current literature to construct such a framework, positioning it as hypothesis-generating by outlining specific, testable predictions to guide future investigation. Based on the literature, we propose that NRDc functions as a crucial molecular indicator and a therapeutic interface, rather than just a bystander enzyme.

## 1 Introduction

Nardilysin (NRDc), alternatively referred to as N-arginine dibasic convertase, functions as a zinc-dependent metalloendopeptidase within the M16 family. It was first identified in the rat brain cortex by Gluschankof et al. in 1984 and initially described for its unique specificity toward dibasic arginine-rich peptides, particularly Arg-Arg motifs (1). Despite being mainly found in the cytoplasm, it can move to the cell surface via a signal-peptide-independent process, indicating a different secretory pathway and a wider variety of biological roles than first thought (2).

At the molecular level, NRDc performs two mechanistically distinct functions. As an intrinsic metalloendopeptidase, it cleaves peptides at N-terminal arginine residues, including direct shedding of VTCN1 (B7-H4) (3). Separately, it activates ADAM-family proteases, which mediate shedding of other substrates such as cytokines and growth factors contributing to immune signaling. Its ability to remodel the proteolytic environment places it at the intersection of metabolic control, inflammation, and cellular homeostasis. The immune system relies on a delicate balance between activation and self-tolerance. NRDc influences this equilibrium by modulating the shedding of cytokines and checkpoint molecules on immune cells. For example, by enhancing ectodomain release of TNF-α through ADAM17, NRDc amplifies pro-inflammatory signaling, and elevated NRDc expression has been observed in the synovial tissue of patients with rheumatoid arthritis (4). Current evidence suggests that dysregulated NRDc activity may amplify autoimmune pathology by reinforcing autoreactive immune signaling and inflammatory cascades, rather than directly initiating disease onset. Its abnormal levels have also been implicated in rheumatoid arthritis (RA), vitiligo, and type 1 diabetes, suggesting its potential as both a therapeutic target and mechanistic contributor (4–6).

Beyond its immunoregulatory role, NRDc is also expressed in neural and metabolic tissues, indicating potential crosstalk between neuro-immune and immuno-metabolic systems. In neuroinflammatory conditions such as multiple sclerosis, its neural functions may modulate immune activity, while in metabolically driven diseases like type 1 diabetes, its metabolic regulation could influence immune balance. This systems-level view aligns with current models that describe autoimmune pathogenesis as network-wide dysregulation rather than a single-pathway defect (7).

A comprehensive literature search was conducted using PubMed and Google Scholar (1984-2026) to identify research on Nardilysin (NRDc). The search utilized keywords including “Nardilysin,” “NRDc,” “immune signaling,” “peptidase substrate specificity,” and “ectodomain shedding,” alongside specific autoimmune conditions such as “Rheumatoid Arthritis,” “Type 1 Diabetes,” and “Vitiligo.” We prioritized literature centered on NRDc’s role in autoimmunity, its biochemical characterization and its normal physiological functions. Studies unrelated to these core topics were excluded. Given the emerging nature of NRDc in clinical research, we compiled available human-derived clinical evidence alongside foundational biochemical and preclinical mechanistic studies to provide a holistic view of the protein’s role in immune homeostasis.

Although the importance of NRDc continues to grow, its precise role in the biological landscape remains to be fully elucidated. This review examines NRDc’s biological and physiological functions and the emerging role in maintaining immune homeostasis, highlighting how its activity provides insight into the early mechanisms of pathogenesis. By analyzing these processes, we identify NRDc as a significant modulator of immune balance and a compelling target for developing novel therapeutic interventions in autoimmunity.

## 2 Gene organization and isoforms

The human NRD1 gene is located on chromosome 1p32.3 and comprises 34 exons, encoding a 1,151-amino-acid protein with a molecular weight of approximately 124 kDa (8). NRDc1 and NRDc2 are the two sequence isoforms produced by alternative splicing (2). They are distinguished by a 68-amino-acid sequence insertion between the acidic stretch and the HXXEH catalytic motif present in NRDc2 (2).

Northern blot analysis revealed that NRDc2 constitutes 50% of total NRDc transcripts in human testis but only 10% in rat (2). Despite the 68-amino-acid insertion in NRDc2, both isoforms exhibit comparable enzymatic properties and inhibitor sensitivity, suggesting conservation of the catalytic core (2). The major difference between these isoforms is the instability of NRDc2, which readily converts to a 110 kDa species under *in vitro* conditions, with cleavage occurring within the 68-aa insertion (2). This cleavage of NRDc was not observed in fresh cellular extracts by western blot analysis, suggesting that this cleavage is unlikely to occur *in vivo* (2).

## 3 Structural and molecular characteristics of Nardilysin

Metallopeptidase family M16 enzymes are among the most abundant peptidases in eukaryotes, bacteria, and archaea. M16 enzymes are structurally grouped into three subfamilies: M16A, M16B, and M16C (9–11). They display structurally similar domains in their quaternary organization. According to the MEROPS classification, NRDc is a zinc-dependent metallo-endopeptidase belonging to the clan ME, specifically family M16 and subfamily M16A. It is classified as an inverzincin because it contains the HXXEH catalytic motif instead of the HEXXH motif that is usually present in metallopeptidases. However, it is distinct from most M16 members by possessing a distinctive 90-amino-acid acidic domain (DAC) near its active site (9). This sequence contains a highly acidic region consisting of 79% glutamic acid and aspartic acid (15). Although mammalian nardilysins have a high degree of overall sequence identity (98% between rat and mouse, 92% between rat and human), conservation in this acidic stretch reduces to 82% and 73% respectively, indicating that it is not essential for the enzyme’s core proteolytic activity (9).

Insulin-degrading enzyme (IDE) is the closest homolog of NRDc, with 38% amino acid sequence identity. The residues responsible for substrate binding and interdomain interactions are highly conserved between IDE and NRDc, suggesting that NRDc likely has a similar clamshell-like structure (12).

## 4 Enzymatic properties and substrate specificity of Nardilysin

NRDc shows optimal activity at pH 8.9, with minor peaks also observed at pH 6.5 (13) or at 6.9 (14), depending on the substrate and buffer conditions. Its metalloendopeptidase nature is confirmed by its inhibition through metal chelating agents like EDTA and 1,10-phenanthroline along with cation inhibition and reactivation profiles. Complete inhibition of NRDc by the thiol-reactive reagent N-ethylmaleimide indicates that one or more cysteine residues are essential for enzymatic activity (13). Amastatin, which is an aminopeptidase inhibitor, inhibits NRDc activity depending on the buffer used, while inhibitors of serine proteases and carboxypeptidases do not affect the enzyme. Because buffer conditions strongly influence NRDc activity, the effect of Tris on the kinetics of peptide hydrolysis was tested (15). Tris, depending on the substrate, increases or decreases the K*M* up to 100-fold, with little effect on V*max*. Other amines, including putrescine, spermidine, and spermine, also affected enzyme activity (15, 16). Spermine did not fully inhibit NRDc even at high concentrations and acted as a noncompetitive inhibitor, indicating that it binds with NRDc at sites other than the active site, specifically spermine was shown to bind directly to an acidic stretch of NRDc. The concentrations required for inhibition are lower than physiological levels, suggesting that its activity may be reduced *in vivo*.

## 5 Extracellular peptide processing and glucagon dynamics

There are several physiological substrates of NRDc. One of them is glucagon (17), and others are the precursors of cytotoxic T lymphocyte epitopes (18). Glucagon is processed into miniglucagon (MG) by MG- generating endopeptidase. MGE containing NRDc and aminopeptidase B cleaves glucagon at Arg17-Arg18 dibasic sites producing mature MG, whose function is opposite to that of glucagon. When NRDc was removed from *α*TC1.6 cell cultures, most of the activity responsible for producing miniglucagon disappeared. This shows that NRDc plays a key role in controlling glucagon processing, suggesting that it’s a regulatory enzyme controlling peptide processing (17).

## 6 HLA class I antigen processing and autoimmune implications

Cytotoxic T lymphocytes (CTLs) recognize short peptide epitopes that are presented on the cell surface by HLA class I molecules. The C-terminal end of these epitopes has been considered to be produced by proteasome activity. However, NRDc has been shown to be required for the generation of several CTL epitopes, including a tumor-specific epitope from PRAME, an immunodominant epitope from the Epstein-Barr virus protein EBNA3C, and a clinically relevant epitope from the melanoma protein MART-1 (18). NRDc contributes to the formation of both the N-terminal and C-terminal ends of these epitopes. It generates these epitopes not only *in vitro* but also in living cells, as reducing its expression using siRNA results in decreased recognition of target cells by epitope-specific CTLs. Analysis of a dataset containing 1,620 ligands from common human HLA class I molecules showed that a dibasic cleavage motif compatible with NRDc activity is present directly at the N-terminus or C-terminus in 10.4% and 4.8% of ligands, respectively. In an additional 5.2% and 5.7% of ligands, this motif is located within four amino acids of the N-terminus or C-terminus. These findings indicate that NRDc can generate precise epitope termini as well as epitope precursors (18).

Because it is involved in HLA class I epitope processing, it may contribute to disease onset in CTL-mediated autoimmune conditions. By acting upstream of TCR signaling and influencing the presentation of self-derived epitopes such as MART-1, altered NRDc activity could increase CTL recognition of normal tissues without directly triggering T-cell activation. However, the physiological importance of NRDc in antigen processing is not yet fully established.

## 7 Cleavage specificity and motif flexibility

Earlier NRDc’s specificity was reported being directed at the N-terminus of an arginine residue within paired basic residues (13). Subsequent studies showed that the exact cleavage position depends on the amino acid preceding the arginine in Xaa-Arg-Lys motifs. When Xaa is His, Phe, Tyr, Asn, or Trp, cleavage occurs exclusively at the Xaa-Arg bond, whereas Ile or Val at this position results in cleavage exclusively between the Arg-Lys residues. For other amino acids, cleavage is observed at both positions to varying extents (19). In addition, it can cleave peptides at a single basic residue when the adjacent P1 residue is hydrophobic, with little difference between arginine and lysine (20). Together, these findings indicate that NRDc shows a preference for basic residues, while the precise cleavage position is determined by local sequence context rather than a fixed cleavage motif. This flexibility helps explain how NRDc acts on mature peptides such as glucagon to generate miniglucagons, and on intracellular proteins to shape HLA class I epitope precursors.

## 8 Subcellular localization, tissue-specific expression, and functional mechanisms

### 8.1 Cytosolic and secretory roles

NRDc is fundamentally a cytosolic enzyme as examined in almost all cell types (2). Alternative translation initiation further contributes to NRDc heterogeneity. HEK293 cells showed the presence of c-myc tag when inserted between Leu28 and Gly-29, indicating that the enzyme can be translated from Met1, which was previously thought to be only initiated from Met49 in rats/mice or Met50 in humans, which generates a protein that lacks this N-terminal signal peptide-like sequence. Although the majority of NRDc is exported via an unconventional secretory pathway, a minor fraction may enter the conventional secretory pathway, explaining the presence of NRDc in secretory granules of α-cells (17), suggesting that the N-terminal region works as a signal peptide in a cell-type-dependent manner. It is also secreted in conditioned medium as in spermatocytes, spermatids (24), and EL-4 cells (14). In the adult rat testis, it is restricted to germ cells, which reach a maximum during late-stage spermiogenesis. Electron microscopy localized NRDc immunoreactivity within the cytoplasm of elongated spermatids, which is specifically localized to microtubular structures like the manchette and axoneme (24), supporting microtubular-dependent processes.

### 8.2 Plasma membrane dynamics

NRDc is also associated with the plasma membrane, determined by immunoprecipitation, cell surface biotinylation, and the heparin-binding epidermal growth factor-like growth factor (HB-EGF) binding capacity on the cell surface (21, 22) to facilitate ectodomain shedding and modulate cell-surface receptor signaling.

### 8.3 Nuclear localization and transcriptional regulation

In NIH3T3 cells, its nuclear expression was localized, where it is found to have a 20-amino acid putative N-terminal nuclear localization signal (NLS). Fluorescence analysis showed 90-95% of NRDc-GFP as cytoplasmic, 3-6% of transfected cells showing both cytosolic and nuclear staining, and 2-4% predominantly showing nuclear staining. Deletion or mutation of the N-terminal NLS showed only cytoplasmic localization, although endogenous NRDc was barely found in the nucleus; its level in the nucleus rose to 15% and 20% after treatment with leptomycin B and spermine, respectively, which shows regulated nuclear shuttling of NRDc (23). It also exhibits cell-cycle-dependent nuclear shuttling in mouse oocytes (25). Beyond this shuttling behavior, NRDc also functions as a transcriptional coregulator by binding to H3K4me2-marked genomic regions (30).

### 8.4 Tissue-specific heterogeneity

NRDc exhibits highly localized and developmentally regulated expression across various tissues. Other than being broadly expressed in all adult rat tissues, it is also distinctly expressed in testis, skeletal muscle, and heart (26, 27). In the central nervous system, it is primarily localized to neurons and dendrites. Its expression is temporally regulated, with significantly higher levels in the early postnatal brain than in the adult brain (28), suggesting a role in early neural development. Furthermore, regional heterogeneity exists while NRDc is robustly expressed in cortical neurons, it remains comparatively low in the basal ganglia and striatum (28, 29), which suggests site-specific functional requirements within the brain.

### 8.5 Biological and physiological roles of NRDc

NRDc binds to dimethylated histone H3K4 and interacts with nuclear proteins such as histone deacetylase 3 (HDAC3), PGC-1α, and Islet-1. Through these interactions, it acts as a transcriptional co-regulator that helps control thermogenesis and glucose metabolism (30). High NRDc levels have been reported in breast, gastric, and esophageal cancers, where it promotes cell growth, although its exact role in cancer development is still unclear (31, 32).

Experiments in Nrdc-knockout mice show increased energy use and poor temperature control, proving its importance in adaptive thermogenesis. During cold exposure, brown adipose tissue (BAT) produces heat through uncoupling protein 1 (UCP1), which is activated by PGC-1α under the control of the sympathetic nervous system (SNS). When NRDc is missing, this pathway is disrupted, suggesting its role in maintaining body temperature and energy balance (33).

In the nervous system, it supports axon growth and myelination by helping release membrane proteins needed for communication between neurons and glial cells. It assists the neuregulin-1 (NRG1) pathway, which is essential for axon signaling and myelin formation. Mice lacking BACE1, an enzyme involved in NRG1 processing, show poor myelination, a problem also seen in Nrdc-knockout mice, linking NRDc to nerve insulation and repair. It also activates ADAM17 (TACE) (34), promoting HB-EGF release (35), improving neural signaling (28). It further regulates cardiac sympathetic nerves by controlling the shedding of the p75 neurotrophin receptor (p75NTR). Mice without NRDc show abnormal nerve patterns in the heart, low blood pressure, and slow heart rate, proving its role in nerve control and heart function (36). Besides these roles, It also influences inflammatory signaling and may help control immune balance, suggesting its potential as a therapeutic target in autoimmune and inflammatory diseases.

## 9 Evolution and expanding functional spectrum of NRDc

Over the past three decades, work on NRDc has moved from basic enzyme characterization to understanding its functions in cell signaling, inflammation, and immune-related diseases. The timeline in **Supplementary Figure 1** outlines how NRDc research expanded from its initial biochemical identification in the 1980s to its current role in development, homeostasis, and autoimmune disease. Early studies clarified its enzymatic activity and receptor interactions, followed by work linking NRDc to TNF-α shedding, neural maturation, transcriptional regulation, and temperature control. More recent findings connect it to autoimmune conditions such as type 1 diabetes, autoimmune arthritis, and vitiligo.

## 10 Mechanistic overview

**Figure 1:**
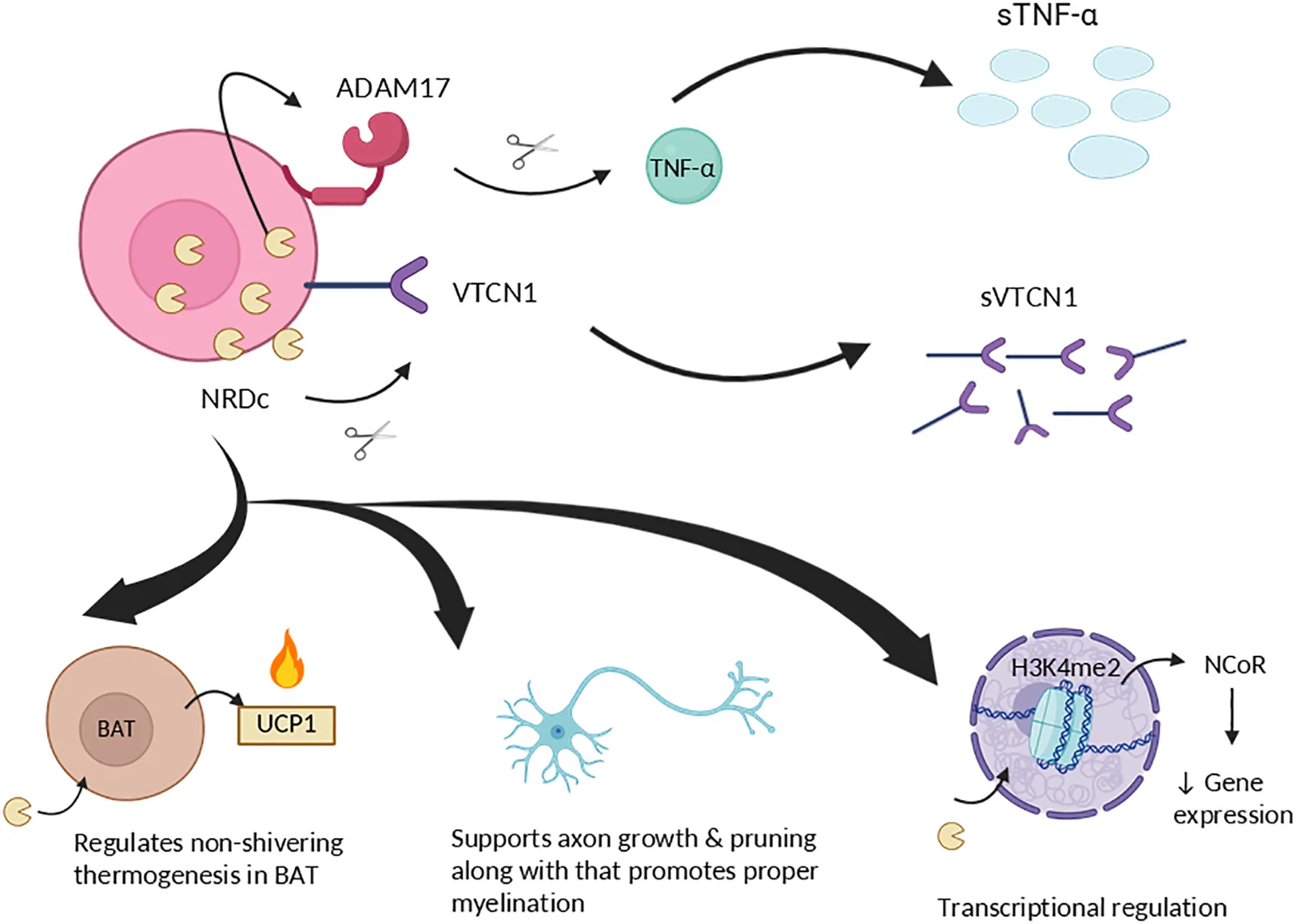
Integrated roles of Nardilysin (NRDc) in ectodomain shedding and key physiological functions. This figure summarizes the multifaceted functions of NRDc. NRDc directly cleaves VTCN1 and facilitates ADAM17-dependent ectodomain shedding of TNF-α, influencing immune regulation. Beyond its immunological actions, NRDc contributes to thermogenesis in brown adipose tissue, supports axonal growth and myelination, and binds H3K4me2 in the nucleus to modulate transcription of selected genes.

As illustrated in **Figure 1**, direct NRDc-mediated cleavage of VTCN1 (B7-H4) promotes autoreactive T-cell proliferation, while NRDc-dependent activation of ADAM17 drives TNF-α shedding and amplifies inflammatory signaling. Together, these parallel but mechanistically independent proteolytic activities position it as a contributor to immune dysregulation and autoimmune pathology.

## 11 NRDc across autoimmune pathologies

NRDc enzyme is known for its shedding or cleavage capacity before the substrate target, such as neuropeptides and growth factors, at the cell surface/free. It is also necessary for various biological processes, such as cell migration, proliferation, and neuroendocrine signaling (28). Recent studies indicate its involvement in regulating the immune system, particularly its potential role in autoimmune diseases.

Understanding the molecular pathways, such as NRD1, that contribute to autoimmunity is crucial for developing effective therapeutic strategies for immune disorders. A delicate balance between stimulatory and inhibitory pathways is maintained in the immune system. That is why dysregulation of these pathways can lead to chronic inflammation and tissue damage. NRDc is emerging as an active participant in immune cell regulation, migration, and catalytic activity, as well as in the production of pro-inflammatory cytokines (21, 34). There is also differential expression of NRD1 in tissues affected by autoimmune diseases.

Recent studies have begun mapping NRDc involvement across systemic and organ-specific autoimmune diseases. **Table 1** summarizes three representative conditions in which NRDc expression, localization, or proteolytic activity is altered and may contribute to.

**Table 1:** Reported NRD1-associated findings in selected autoimmune diseases.

No.	Autoimmune diseases	Therapeutic usage/Functional finding involving NRD1	Technical details	Reference
1.	Type 1 diabetes	Blocking NRD1 activity stabilizes membrane VTCN1 and reduces antigen-specific T-cell proliferation.	NRD1 inhibitors (phenanthroline, amastatin, bestatin) and NRD1-siRNA were used; inhibition reduced sVTCN1 release and kept VTCN1 on the cell surface; reduced G9C8 T-cell proliferation.	Radichev et al., 2014 (5)
2.	Rheumatoid Arthritis	Reducing NRD1 activity (genetic or siRNA) lowers arthritis severity by decreasing TNF-α shedding.	NRD1 knockout mice and anti-NRD1 siRNA in arthritis models reduced inflammation and TNF-α release; increased NRD1 seen in RA synovial environments.	Fujii et al., 2017 (4)
3.	Vitiligo	Altered NRD1 expression is observed in patients; suggests involvement in disease pathogenesis.	Increased NRD1 transcript in skin and PBMCs, increased NRD1 protein in plasma, and altered VTCN1 levels in vitiligo patients.	Vaishnav et al., 2022 (6)

This table summarizes reported roles of NRD1 in selected autoimmune diseases. For each condition, key functional findings or therapeutic implications are listed along with the experimental systems and interventions used to establish NRD1 involvement. References correspond to primary research articles reporting these outcomes.

### 11.1 Nardilysin in type 1 diabetes

Type 1 diabetes (T1D) is an autoimmune disease in which the immune system selectively destroys insulin-producing β-cells in the pancreatic islets, resulting in lifelong insulin dependence and continuous metabolic monitoring. The key immune contributors of T1D include autoreactive CD8^+^ and CD4^+^ T cells, impaired regulatory T-cell (Treg) control, and increased pro-inflammatory cytokines such as TNF-α and IL-1β (37). Although some autoreactive T cells normally escape thymic negative selection, these cells typically remain naïve in healthy individuals (38, 39). In T1D, this tolerance mechanism no longer works properly, so islet-specific T cells that usually stay quiet become activated, marking a critical step in β-cell destruction (39). Under normal conditions, APCs must deliver both an antigen-specific signal and a costimulatory signal for T-cell activation. If either of these signals becomes excessive, insufficient, or poorly controlled, T cells can activate in the wrong context which leads to damaging autoimmune responses (40).

VTCN1 (also known as B7-H4, B7S1, or B7X) is an inhibitory costimulatory molecule that normally limits T-cell activation (41). It signals through an unidentified receptor on T cells and suppresses downstream pathways involving ERK, JNK, and Akt (42). Experimental studies support its protective role in T1D, as VTCN1-Ig inhibits the proliferation of islet-specific T-cell clones from T1D patients, and overexpression of VTCN1 in human and mouse islets similarly reduces their susceptibility to T-cell-mediated cytotoxicity. This pattern is further seen *in vivo*, where β-cell-specific VTCN1 overexpression in NOD mice protects against diabetes induced by both CD4^+^ and CD8^+^ islet-reactive T-cell clones (5). These findings indicate that reduced VTCN1-mediated inhibition contributes to abnormal activation of diabetogenic T cells. NRDc regulates this pathway by cleaving membrane-bound VTCN1 to generate soluble VTCN1 (sVTCN1), which lacks inhibitory function (5). Protease-inhibition and gene-silencing experiments identified NRDc as a major enzyme responsible for this cleavage in APCs. T1D patients show increased NRDc expression in peripheral blood mononuclear cells (PBMCs) and elevated serum sVTCN1 levels, indicating enhanced VTCN1 shedding during disease progression. Similar findings in NOD mice support this mechanism. The strong correlation between NRDc levels and sVTCN1 release suggests that excessive cleavage of VTCN1 weakens negative costimulation and facilitates expansion of autoreactive T cells. Importantly, elevated sVTCN1 is already detectable in new-onset T1D, indicating that NRDc-mediated VTCN1 loss begins early in the disease course (5).

Overall, current evidence indicates that NRDc does not initiate T1D but can intensify autoimmune activity by inactivating VTCN1 once tolerance is broken. The early rise in NRDc and sVTCN1 levels highlights their potential utility as early indicators of progressing β-cell autoimmunity. Targeting NRDc-mediated VTCN1 cleavage may also offer a therapeutic strategy for maintaining immune regulation in individuals at risk for T1D.

### 11.2 Nardilysin in rheumatoid arthritis

Rheumatoid arthritis (RA) is a chronic inflammatory disease marked by persistent synovial inflammation, joint damage, and pain, affecting approximately 0.2–1% of the global population (43, 44). A key driver of RA pathology is tumor necrosis factor-α (TNF-α), which is highly expressed in synovial tissues and predominantly produced by macrophages. Cytokines and chemokines released in the synovium recruit B cells, T cells, and neutrophils and promote additional cytokine release, matrix metalloproteinase activity, and osteoclastogenesis, which leads to joint inflammation and bone erosion. Due to the presence of auto-antibodies like rheumatoid factor (RF) and anti-citrullinated peptide antibodies (ACPA), loss of self-tolerance occurs early and can be detected years before clinical arthritis (45, 46). These autoantibodies contribute to tissue injury by forming immune complexes in synovial tissues, as demonstrated in autoantibody-induced arthritis (AAIA) models, which include collagen antibody-induced arthritis (CAIA) and K/BxN serum-transfer arthritis (K/BxN STA).

TNF-α is central in both CAIA and K/BxN STA models (46–49). TNF-α is synthesized as a membrane-anchored precursor and requires ectodomain shedding by ADAM17 to become biologically active. While anti-TNF-α therapy helps with CAIA, it is ineffective in K/BxN STA, highlighting model-specific differences in TNF-α dependence. Studies comparing TNF-α-null mice and mice expressing a non-cleavable form of TNF-α show that soluble TNF-α is dispensable for lymphoid organogenesis but essential for optimal inflammatory lesion development, underscoring the importance of ADAM17-dependent TNF-α shedding in inflammatory disease (47). Supporting this, myeloid-specific ADAM17-deficient mice and mice lacking iRHOM2 (required for ADAM17 maturation), are protected from K/BxN STA, confirming that ADAM17-mediated TNF-α cleavage is necessary for autoimmune arthritis (48, 49).

NRDc has been shown to bind directly to ADAM17 and increase its catalytic activity, and this enhancement occurs independently of its own peptidase function (35). Experimental studies using bone marrow-derived macrophages (BMDMs), peritoneal macrophages (PMs), and THP-1 cells demonstrate that loss of NRDc reduces the amount of cleaved/soluble TNF-α released by these cells, while TNF-α mRNA levels remain unchanged. NRDc loss in these systems was achieved through genetic deletion in mice and by targeted RNA interference in THP-1 cells. *In vivo*, intra-articular anti-Nrdc siRNA similarly lowered TNF-α shedding and reduced disease severity in CAIA mice (4).

This indicates that it does not regulate TNF-α transcription but instead modulates TNF-α post-translationally, specifically by promoting ADAM17-mediated ectodomain shedding at the cell surface. Through this mechanism, it facilitates increased release of active TNF-α and contributes to the inflammatory processes, a characteristic of autoimmune arthritis. Clinically, RA patients exhibit significantly higher synovial levels of both NRDc and TNF-α compared to individuals with osteoarthritis (OA), and their levels correlate with TNF-α and serum C-reactive protein (CRP), suggesting that elevated NRDc may enhance local TNF-α production and worsen joint inflammation (4). Interestingly, NRD1 also appears among the 101 upregulated genes in an independent transcriptomic study of peripheral blood mononuclear cells from RA patients, though the authors did not comment on it directly, nor was it among the genes selected for qPCR validation (50). This suggests that elevated NRDc expression in RA is not confined to synovial tissue but extends into the peripheral circulation as well.

Therapeutically, NRDc inhibition shows clear potential. Intra-articular anti-NRDc siRNA reduces disease severity in collagen antibody-induced arthritis (CAIA) mice. Unlike standard TNF-α blockers, which suppress both pathological and normal TNF-α activity and increase infection risk, NRDc deletion does not affect baseline TNF-α production in macrophages and monocytes (4). It mainly limits the excess ADAM17-dependent TNF-α shedding during inflammation, making it a more selective target for controlling arthritis without disrupting normal immune defense.

This suggests that NRDc inhibition may selectively reduce pathological TNF-α shedding while preserving essential immune functions. ADAM17 is another potential target, but systemic ADAM17 inhibition affects multiple substrates, including EGF-receptor ligands and Notch, raising concerns about adverse effects on the skin, liver, and intestines (35). Collectively, these findings position it as a contributor to RA pathogenesis through its enhancement of ADAM17-mediated TNF-α shedding and as a potential therapeutic target that may allow more precise modulation of inflammatory signaling than current TNF-α-blocking strategies.

### 11.3 Nardilysin in vitiligo

Vitiligo is an autoimmune disorder in which the T cells damage the melanocytes, causing gradual loss of skin pigment. Globally, it affects about 0.5–1% of the population, wherein 50% of the cases show occurrence around the age of 20, and all genders are affected equally (51). Vitiligo causes melanocyte damage on a major scale.

Thorough investigation has shown changes in signaling pathways in vitiligo patients, along with overactivation of mitogen-activated protein kinase (MAPK) and potentially due to long-term oxidative stress, creating an elevated susceptibility for apoptosis. These melanocytes also produce increased levels of IL-6, MMP-3, IGFBP-3, and IGFBP-7 (52, 53). It leads to both antibody-mediated and T-cell-mediated responses. Patients show the presence of melanocyte-specific autoantibodies, autoreactive CD8^+^ T cells in the skin, and reduced Treg activity (54). Co-stimulatory molecules also influence this loss of tolerance. The B7–CD28 family controls T-cell activation (55). Within this family, VTCN1 (B7-H4/B7S1/B7x) acts as an inhibitory molecule that limits T-cell activation, clonal expansion, and cytokine release (41, 56).

NRD1 cleaves the membrane-bound VTCN1 on the antigen cells, creating a soluble VTCN1 (sVTCN1) which no longer functions as an inhibitor (6). This cut removes a barrier to T cell activity. Investigations in vitiligo patients show a stable and positive connection between the levels of the NRD1 protein and the sVTCN1 concentration in the bloodstream. Other evidence shows reduced CD4^+^/CD8^+^ ratios, Tregs, cytokine imbalance, oxidative stress, and ER stress. Vitiligo patients show a significant increase in the presence of NRD1 transcripts in the blood and skin. Due to the cleaving of VTCN1 by NRD1, the circulation of sVTCN1 increases. A study showed a difference between early-onset (≤3 months) and late onset vitiligo, where early cases had high NRD1 and sVTCN1 levels, suggesting NRD1 level rises early in the disease. Increased NRD1 in PBMCs and skin potentially increases the loss of the membrane-bound VTCN1, reducing T cell inhibition (6). Hence, NRD1-led reduction of VTCN1 could weaken the inhibitory signals on the T cells, encouraging a stronger auto-reactive response against the melanocytes and contributing to the progression of the disease in the patient.

## 12 NRDc inhibition as a therapeutic strategy: what current data show

Multiple studies show that NRDc can be used as a therapeutic target because of how it affects TNF-α shedding and VTCN1 cleavage. The first clear evidence came from its knockdown in THP-1 monocytic cells using lentiviral RNAi. Once its mRNA and protein levels dropped, LPS-stimulated TNF-α in the supernatant went down as well. But TNF-α mRNA and the intracellular protein didn’t change (4). So, it wasn’t controlling TNF-α production; rather it was mainly affecting how much of it got cleaved and released. Mouse macrophages showed the same pattern as without NRDc, they produced less active cleaved TNF-α after stimulation.

To see if this mattered in a disease model, researchers looked at immune-complex-driven TNF-α release. Out of three siRNAs, one (siRNA3) worked best and was tested *in vivo.* In CAIA mice, this siRNA reduced arthritis severity during days 3–8 and 12–14 (4). Another study used its inhibitor 1,10-phenanthroline, which lowered sVTCN1 release from macrophages and 3T3-flVTCN1 (full-length VTCN1) cells (5).

These findings suggest two angles for therapy which are limiting pathological TNF-α shedding or reducing VTCN1 cleavage. Both approaches could help restore immune balance, though the challenge remains to target NRDc without affecting other M16 family functions (4, 5).

## 13 Discussion

**Figure 2:**
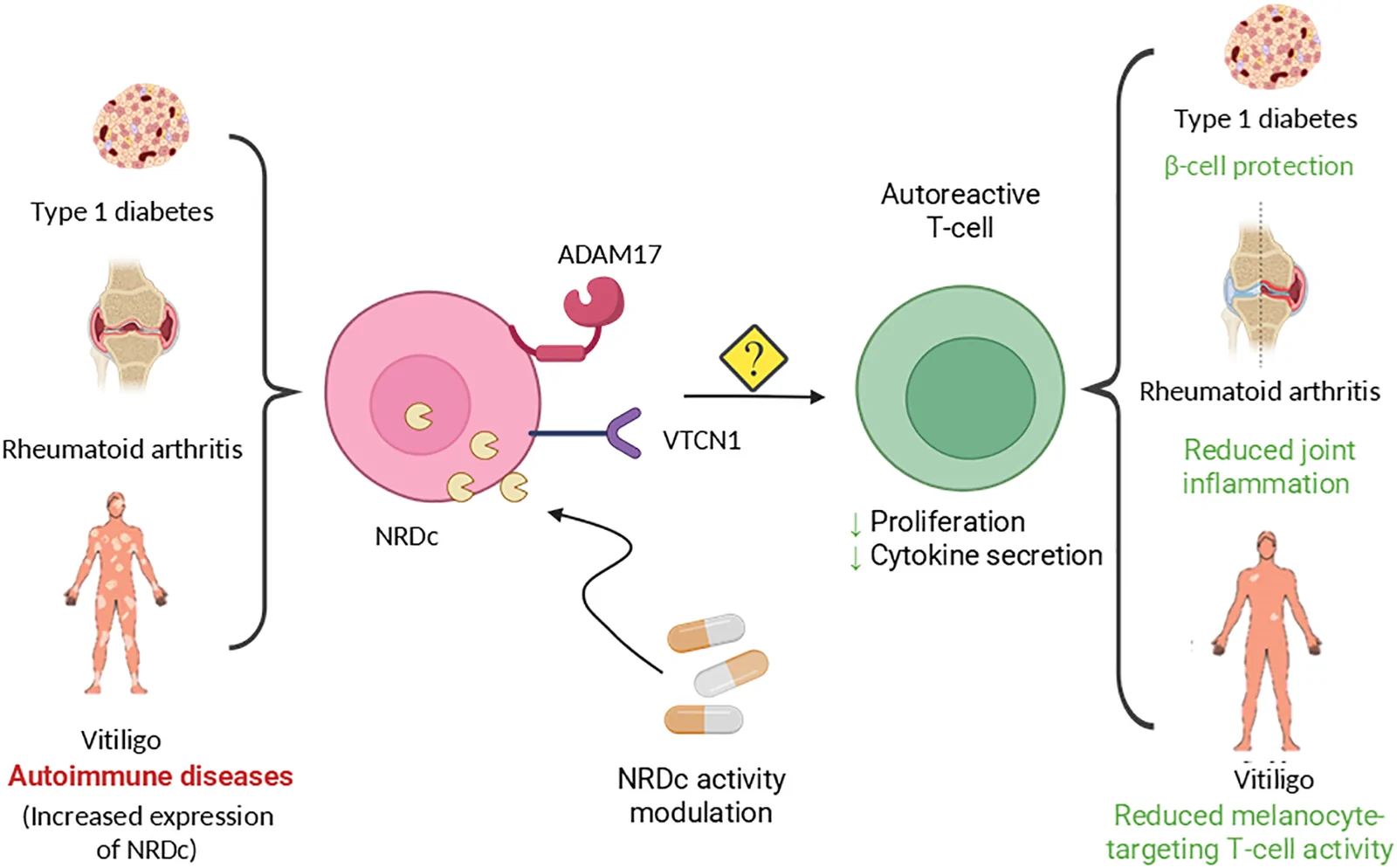
NRDc modulation as a strategy to restore immune balance in autoimmune diseases. The proposed mechanism is that modulation of NRDc activity lowers T-cell proliferation and cytokine release, leading to disease-relevant improvements including β-cell protection, reduced joint inflammation, and decreased melanocyte-directed immune responses.

NRDc has several physiological roles, but its involvement in immune regulation is particularly significant. It has been implicated in HLA class I epitope processing, including a tumor-specific epitope from PRAME, an immunodominant epitope from the Epstein-Barr virus protein EBNA3C, and a clinically relevant epitope from the melanoma protein MART-1 which suggests that altered NRDc activity could increase CTL recognition of normal tissue without directly triggering T-cell activation, and that it may contribute to disease onset. Beyond epitope processing, NRDc influences immune activity through distinct proteolytic roles at the cell surface. In rheumatoid arthritis, it enhances ADAM17-dependent TNF-α shedding, whereas in type 1 diabetes and vitiligo, it contributes to the cleavage of membrane-bound VTCN1. Although these represent different molecular substrates, the outcome across all three conditions is a reduction in inhibitory control within T-cells, facilitating an exaggerated immune response. As illustrated in **Figure 2**, these pathways position NRDc as a potential therapeutic target. While our current understanding is derived primarily from these three models, NRDc’s distinct proteolytic activities suggest it may play a similar role in other autoimmune contexts. Taken together, these mechanisms offer a framework for understanding how NRDc contributes to disease pathogenesis.

This framework is nonetheless based on a limited body of evidence. The mechanistic understanding of NRDc in type 1 diabetes relies heavily on a single primary reference, and the selection of rheumatoid arthritis, type 1 diabetes, and vitiligo reflects the current availability of literature rather than an exhaustive survey of autoimmune conditions more broadly. While elevated blood sVTCN1 levels have been identified as a potential indicator of disease progression and severity in T1D, pointing to its possible use as a biomarker, its broader clinical application in other autoimmune diseases remains hypothesis-generating in the absence of direct experimental validation. Finally, the specific cellular sources and inflammatory pathways that regulate NRDc expression remain unidentified, and it is not yet known whether elevated NRDc levels actively drive disease or simply reflect ongoing inflammation.

Regarding therapeutic potential, studies indicate that reducing NRDc activity can limit VTCN1 shedding or excess cytokine release. However, NRDc also supports normal function in other tissues as particularly, in pancreatic α-cells, where it is required for processing glucagon into miniglucagon, a peptide with activity opposite to that of glucagon itself. This matters because the pancreatic islet is also the proposed site of NRDc’s pathological role in T1D, so systemic inhibition risks disrupting normal endocrine function in the very tissue implicated in disease. Systemic inhibition is therefore not a viable strategy; future approaches must instead find ways to modulate NRDc activity specifically where it is pathologically elevated. Given the growing interest in CAR-T cell therapy and targeted biologics for autoimmune disease, NRDc’s role across these three conditions makes it worth considering as a similar kind of target, provided this depends on finding ways to act locally rather than systemically.

## 14 Conclusion and future direction

NRDc is clearly involved in the modulation of immune responses, though the full extent of its role remains to be established. Current evidence suggests that NRDc-mediated proteolysis is a common feature across select autoimmune conditions, but further work is required to distinguish its specific triggers in each disease. Future research should prioritize identifying which domains of NRDc are responsible for its immune-related effects to determine if they can be modulated without disrupting its normal physiological functions. Moving beyond initial hypotheses will require defining the specific cellular pathways that govern its expression. Uncovering these details holds great promise, as refining our understanding of this enzyme offers an exciting path toward developing more precise, targeted strategies for the next generation of autoimmune care.

## Glossary

AAIA: Anti-arthritogenic antibody
ACPA: Anti-citrullinated protein antibody
ADAM17: A disintegrin and metalloproteinase 17
Akt: Protein kinase B
APCs: Antigen-presenting cells
αTC1.6: Mouse pancreatic alpha-cell line
BACE1: Beta-site amyloid precursor protein cleaving enzyme 1
BAT: Brown adipose tissue
BMDMs: Bone marrow-derived macrophages
CAIA: Collagen antibody-induced arthritis
CRP: C-reactive protein
CTLs: Cytotoxic T lymphocytes
c-myc: Cellular myelocytomatosis oncogene
EBNA3C: Epstein-Barr virus nuclear antigen 3C
EDTA: Ethylenediaminetetraacetic acid
ERK: Extracellular signal-regulated kinase
H3K4me2: Histone H3 lysine 4 dimethylation
HB-EGF: Heparin-binding epidermal growth factor-like growth factor
HDAC3: Histone deacetylase 3
HEK293: Human embryonic kidney 293 cells
HLA: Human leukocyte antigen
IGFBP: Insulin-like growth factor-binding protein
JNK: c-Jun N-terminal kinase
K/BxN STA: K/BxN serum transfer arthritis
LPS: Lipopolysaccharide
MAPK: Mitogen-activated protein kinase
MART-1: Melanoma antigen recognized by T cells 1
MEROPS: Database of peptidases and their inhibitors
MG: Miniglucagon
MGE: Miniglucagon-generating endopeptidase
MMP-3: Matrix metalloproteinase-3
NIH3T3: Mouse fibroblast cell line
NLS: Nuclear localization signal
NOD: Non-obese diabetic mouse model
NRDc: Nardilysin
NRG1: Neuregulin 1
OA: Osteoarthritis
p75NTR: p75 neurotrophin receptor
PBMCs: Peripheral blood mononuclear cells
PGC-1α: Peroxisome proliferator-activated receptor gamma coactivator 1-alpha
PMs: Peritoneal macrophages
PRAME: Preferentially expressed antigen in melanoma
TACE: Tumor necrosis factor-alpha converting enzyme
TCR: T-cell receptor
THP-1: Human monocytic cell line
TNF-α: Tumor necrosis factor-alpha
UCP1: Uncoupling protein 1
VTCN1: V-set domain-containing T-cell activation inhibitor 1
